# Prediction of protein secondary structure by the improved TCN-BiLSTM-MHA model with knowledge distillation

**DOI:** 10.1038/s41598-024-67403-0

**Published:** 2024-07-17

**Authors:** Lufei Zhao, Jingyi Li, Weiqiang Zhan, Xuchu Jiang, Biao Zhang

**Affiliations:** 1 Agricultural Science and Engineering School, Liaocheng University, Liaocheng, 252059 China; 2 School of Statistics and Mathematics, Zhongnan University of Economics and Law, Wuhan, 430073 China; 3 Emergency Management Research Center, Zhongnan University of Economics and Law, Wuhan, 430073 China; 4 School of Computer Science, Liaocheng University, Liaocheng, 252059 China

**Keywords:** Temporal convolutional network (TCN), Knowledge distillation, Protein structure prediction, Computational biology and bioinformatics, Engineering, Mathematics and computing

## Abstract

Secondary structure prediction is a key step in understanding protein function and biological properties and is highly important in the fields of new drug development, disease treatment, bioengineering, etc. Accurately predicting the secondary structure of proteins helps to reveal how proteins are folded and how they function in cells. The application of deep learning models in protein structure prediction is particularly important because of their ability to process complex sequence information and extract meaningful patterns and features, thus significantly improving the accuracy and efficiency of prediction. In this study, a combined model integrating an improved temporal convolutional network (TCN), bidirectional long short-term memory (BiLSTM), and a multi-head attention (MHA) mechanism is proposed to enhance the accuracy of protein prediction in both eight-state and three-state structures. One-hot encoding features and word vector representations of physicochemical properties are incorporated. A significant emphasis is placed on knowledge distillation techniques utilizing the ProtT5 pretrained model, leading to performance improvements. The improved TCN, achieved through multiscale fusion and bidirectional operations, allows for better extraction of amino acid sequence features than traditional TCN models. The model demonstrated excellent prediction performance on multiple datasets. For the TS115, CB513 and PDB (2018–2020) datasets, the prediction accuracy of the eight-state structure of the six datasets in this paper reached 88.2%, 84.9%, and 95.3%, respectively, and the prediction accuracy of the three-state structure reached 91.3%, 90.3%, and 96.8%, respectively. This study not only improves the accuracy of protein secondary structure prediction but also provides an important tool for understanding protein structure and function, which is particularly applicable to resource-constrained contexts and provides a valuable tool for understanding protein structure and function.

## Introduction

Methods for predicting and designing protein structures have made tremendous progress over the past decade. Increases in computing power and the rapid growth of protein sequence and structure databases have driven the development of new data-intensive and computationally demanding structure prediction methods^1^. In recent years, significant technological progress has been achieved in the field of protein structure prediction; these methods have surpassed previous methods that rely on a single protein sequence or structural model, and cutting-edge technologies such as Alphafold2 and RoseTTAFold^2^ have also been developed. These technologies have achieved unprecedented achievements in protein structure prediction with the help of the powerful capabilities of deep learning. These transformative developments indicate that artificial intelligence will play an even more critical role in future biomedical research and applications.

This study introduces a novel temporal convolutional network (TCN) layer and its derived composite model, “improved TCN-BiLSTM-MHA,” which integrates a TCN, bidirectional long short-term memory (BiLSTM) networks, and a multi-head attention mechanism (MHA). The protein sequences are tokenized using the word2vec method, and one-hot encoding features and the physicochemical properties of the protein sequences are extracted to obtain concatenated word vectors. Additionally, utilizing knowledge distillation, the study facilitated the student model to learn richer features from the ProtT5-XL-UniRef teacher model. The model predicts the eight-state and three-state structures of proteins in classic datasets such as TS115, CB513^3^, CASP13, CASP14, and CASP15 and predicts the eight-state and three-state structure data in the PDB protein database^4^ from June 6, 2018, to 2020. The training sets of the TS115, CB513, and PDB (2018–2020) protein databases were derived from a random division of their own datasets at a ratio of 7:3, and the training set of the PDB protein database was derived from the PDB data intercepted in this paper. The prediction accuracy of the eight-state structure of the six datasets in this paper reached 88.2%, 84.9%, and 95.3%, and the prediction accuracy of the three-state structure reached 91.3%, 90.3%, and 96.8%, respectively.

The main contributions of this article include (1) proposing an improved TCN model and optimizing the structure of the TCN layer; (2) combining word2vec tokenization with the improved TCN-BiLSTM-MHA framework to enhance the predictive performance of the protein secondary structure; and (3) employing a protein language model for knowledge distillation to achieve superior prediction results in smaller model configurations.

### Literature review

Machine learning techniques play a central role in the field of protein structure prediction. Numerous protein data are used to train models to learn and extract information to predict protein structure and function. Currently, widely used methods can be divided into two main categories: machine learning methods and deep learning methods based on amino acid sequences. Machine learning prediction methods for amino acid sequences include algorithms such as random forest (RF), extreme gradient boosting (XGBoost) and the naive Bayes network^5^, while machine learning prediction methods such as convolutional neural networks (CNNs), residual networks (ResNets), and recurrent neural networks (RNNs) have been widely used for protein structure prediction^6^. These machine learning and deep learning models can automatically identify and extract features from protein data to more accurately predict protein structure and function. At the same time, these methods predict protein structure and function by converting amino acid sequences into numerical vectors and applying feature extraction techniques. Structure and function. To enhance the performance of the prediction model, multiple coding methods are combined, such as PSSM coding, one-hot coding and HMM coding^7^, and multiple methods are used to combine the coding.

Machine learning and deep learning technologies have made remarkable achievements in protein structure prediction, providing powerful tools to analyze and predict protein structure and function, but these methods still have some shortcomings. First, although these models can automatically extract features and make predictions, their accuracy still needs to be improved when dealing with nonhomologous sequences or proteins with highly complex structures. Second, deep learning models usually require a large amount of training data to achieve optimal performance, and high-quality protein structure data are often difficult to obtain, which limits the training and generalization capabilities of the models. In addition, the interpretability of deep learning models is also a challenge, and the decision-making process of the model is usually regarded as a “black box”, which is a shortcoming for biological and medical research that requires a clear understanding of the basis for model predictions. At the same time, the training and inference process of the model often requires a large amount of computing resources, which may be a constraint in resource-limited environments.

In the field of computational biology, natural language processing technology, especially protein language models (PLMs), has rapidly demonstrated its application value. This type of model translates protein sequences into rich and high-dimensional representations, enabling them to perceive sequence content, and then be applied to protein folding classification, function prediction, and structure prediction and design. Currently, commonly used protein language models have been proven to be powerful tools for sequence design, mutation effect prediction, and structure prediction^8^. Commonly used protein language models include ProtBERT, evolutionary scale modeling (ESM), and Alphafold.

Protein language models have attracted attention due to their ability to capture the deep features of proteins during their pretraining process. By combining machine learning and deep learning technologies, these models can analyze the interaction network between amino acid sequences and proteins to achieve functional prediction. Currently, the application of protein language models has been extended to various protein families to improve the controllability of their generation performance. The latest research shows that protein language models can be widely used in a large number of fields, can improve the computational efficiency of research, and have strong practicability^9^.

Protein language models have shown significant advantages in computational biology, being able to convert protein sequences into high-dimensional representations and capture deep features, and are widely used in the prediction of protein structure and function. These models leverage advanced machine learning techniques to increase the computational efficiency and application scope of research. However, the effectiveness of PLM depends on the quality of the training data, its complex decision-making process may affect the interpretability of the results, and its performance may be limited under certain specific conditions. For example, models such as Alphafold2 have made certain achievements in protein secondary structure prediction, but their prediction capabilities are still limited for proteins with low homology^10^. Nonetheless, PLM provides powerful tools and new perspectives for the future development of protein science.

Knowledge distillation was first introduced by Hinton et al.^11^, primarily involving the transfer of knowledge from a complex teacher model to a simpler student model to enhance the student model's performance. This technique has shown potential in protein structure prediction. However, there are challenges in practical applications, such as the performance gap between the teacher and student models, which might affect the learning outcomes of the student model. Current research on knowledge distillation mainly focuses on improving loss calculation methods^12^. There is a need to develop more effective model compression and knowledge distillation strategies to reduce the computational resource requirements of high-performance models while expanding their applications and integrating with other learning schemes^13^. Furthermore, exploring the fusion of protein language models and knowledge distillation is a key future direction. This approach can fully leverage the features learned from large datasets via protein language models, improving the accuracy and efficiency of structure prediction. For instance, Wang et al.^14^’s Contact-Distil technique introduces knowledge distillation into protein structure prediction, enhancing not only the predictive performance but also the accuracy of low-homology protein contact map prediction (PCMP).

The application of knowledge distillation techniques in protein language models has demonstrated advantages in enhancing model performance and reducing computational resource demands, especially in improving the accuracy of protein structure prediction. Key steps to comprehensively enhance the performance and application scope of protein structure prediction include in-depth research and optimization of model compression and knowledge distillation methods. This encompasses developing more efficient and lightweight distillation strategies for resource-intensive advanced models such as AlphaFold2, reducing the demand for computation and memory, and enhancing its applicability to standard hardware. It also involves exploring how to effectively integrate protein language models with other prediction models. Through such integration, the features extracted from large datasets by protein language models can be maximized in combination with traditional models, thereby improving the accuracy and efficiency of structure prediction. Moreover, multidimensional optimization of protein structure prediction should not only focus on improving existing models such as AlphaFold2 but also include the distillation of other models and the integrated application of different feature sets to achieve a comprehensive enhancement of protein structure prediction techniques.

### Advanced algorithms

This chapter reviews the currently popular prediction directions for protein language modeling. In this section, the latest algorithms for protein secondary structure prediction are systematically described, as shown in Table 1:

**Table 1:** Latest prediction algorithms for the prediction of protein secondary structure.

Models	Method	Advantages	Disadvantages
CNN-LSTM^15^	Extracting features of protein sequences based on a fused form of CNN and LSTM	Uses a widely used deep learning methodology with obvious results and a simple structure	Simple model structure and low accuracy of results
Transformer^16^	Protein structure prediction using the Transformer self-attention mechanism model	The effect is obvious and is the base model for most protein language models	Transformer models can be complex, which can make understanding and tuning the algorithm difficult
ProteinGCN^17^	A protein topology map was created using one-hot vector coding as features to represent atoms as nodes	In Sanyal et al.^17^ study, ProteinGCN is more suitable for protein structure prediction than 3D-CNN model	Still using a shallow architecture, it is not possible to fully extract the protein structure and get the best results
Alphafold2^2^	Utilizing Evoformer neural network blocks as a means of processing the input data and generating a processed representation of multiple sequence pairs and residue pairs	AlphaFold2 has amazing accuracy and is one of the most accurate prediction models available	AlphaFold2 requires strong sequence homology, relies too heavily on multiple sequence comparison (MSA), and is more limited in its application, possessing drawbacks such as difficulty in predicting intrinsically disordered proteins/regions and loops^10^
ESMFold^18^	The Transformer architecture was used, trained by modeling a masked language on a large number of protein sequences	ESMFold replaces MSA with a language model that generates a single sequence of features, avoiding Alphafold2’s overreliance on MSA while dramatically increasing runtime speed	ESMFold still does not handle specific types of protein structures well, and furthermore, while the model has made progress in predicting single-sequence protein structures, there is still room for improvement in dealing with multiple sequence alignments and template dependencies
RoseTTAFold^19^	An attention mechanism with structural bias is employed using protein 3D parallel module information, as well as Alphafold2's frame comparison module, FAPE loss function and data distillation techniques	RoseTTAFold2 is a single model with an accuracy equivalent to that of AF2 for monomers and AF2 multimers for complexes, and has better computational scaling for proteins and complexes larger than 1000 residues	The model structure is more complex, and the overall accuracy of Alphafold2 is higher than that of RoseTTAFold
Rgn2^20^	A protein structure prediction method based on deep learning and protein language modeling (AminoBERT). AminoBERT is used to capture potential structural information in protein sequences and to describe the geometrical structure of proteins by means of the Frenet-Serret formula	RGN2 outperforms AlphaFold2 and RoseTTAFold in orphan and designer protein classes, while reducing computation time by up to a factor of 10^6^ times	The model has some challenges in predicting proteins rich in β-folded structures and the fine-tuned part of RGN2 has not been fully integrated into the end-to-end implementation in the current version of the implementation. These limitations may affect the performance of the model in specific applications

As Table 1 illustrates, in the field of protein secondary structure prediction, the latest advancements in deep learning demonstrate the integrated application of various advanced models, including convolutional neural networks, Transformer models, and graph neural networks. This trend highlights the deepening application and development of deep learning technologies in this area.

AlphaFold2, one of the most noteworthy models in recent years, is renowned for its exceptional predictive accuracy and stability. Despite its strengths, it relies heavily on multiple sequence alignment (MSA), which restricts its broader application. To address this issue, researchers have developed new models, such as Rgn2, which can effectively predict the secondary structure of isolated protein sequences that have not undergone MSA. Furthermore, studies have shown that protein language models such as ESMfold also excel in tackling similar issues, demonstrating superior model performance.

Nevertheless, these advanced prediction methods face common challenges, such as the demand for high computational resources and limitations in handling specific protein structures. Despite these challenges, protein language models and their related application fields are receiving the attention of researchers, who are encouraging them to explore alternative models with broader applicability to overcome the limitations of AlphaFold2.

## Related work

This chapter provides a literature review on deep learning for protein secondary structure prediction problems, protein language modeling, and distillation learning.

In the field of deep learning, Xu et al.^21^ utilized the ResNet model, which significantly enhanced the results of CASP13. Compared to nondeep learning methods based on coevolution in CASP13 and other deep learning models not based on coevolution (such as the popular recurrent geometric network, RGN), this model demonstrated notable advancements. Additionally, Dong et al.^22^ employed mechanisms such as dilated convolution (D-Conv) and the squeeze-and-excitation network (SENet) for local feature extraction and target channel enhancement. Next, they utilized a combination of recurrent neural network variants (BiGRU and BiLSTM) with transformer modules to achieve global bidirectional information consideration and feature enhancement. With the increasing popularity of transformers, some scholars have also attempted to apply them to protein secondary structure prediction, effectively extracting structural features between amino acid sequences, as seen in protein language models such as AlphaFold2, which are based on modifications of the Transformer. Zhou et al.^23^ leveraged the strengths of CNNs and transformers to improve the quality of sequence embeddings and used knowledge distillation to enhance a multiscale network, further improving the prediction accuracy and efficiency. However, the TCN model has also been widely applied. Zhang et al.^24^ combined convolutional neural networks (CNNs) with bidirectional TCNs to predict protein secondary structures. The model's advantage lies in its ability to effectively extract local features of amino acid sequences and analyze long-distance interactions within them, thereby enhancing the accuracy of protein secondary structure prediction. Yuan et al.^25^ also employed the TCN model for feature extraction from protein sequences and achieved positive results. However, TCNs can only extract unidirectional features. Secondary structure prediction is influenced by both past and future amino acids, and the function and structure of a protein are determined by its multidimensional spatial structure. Transmitting information in only one dimension is insufficient. The introduction of multidimensional bidirectional information not only considers the anterior and posterior information of amino acid sequences but also captures multidimensional relationships. Longer individual protein sequences require more precise modeling to capture long-term dependencies.

In the domain of protein encoding and prediction, protein language models have been extensively utilized. Ferruz et al.^26^ designed ProtGPT2 by training approximately 50 million unlabeled protein sequences. Predictions made using AlphaFold2 indicated that the sequences generated by ProtGPT2 could form well-structured, nonidealized three-dimensional structures. Weissenow et al.^27^ employed a model named EMBER2, which is based on embeddings from pretrained protein language models (pLMs), to predict 2D residue distance structures between proteins, specifically using attention head embeddings from the ProtT5 model. The advantage of EMIBER2 lies in its independence from multiple sequence alignments (MSAs), allowing for structure predictions at a lower computational cost and faster speed, thereby enhancing computational efficiency in predictions. For example, Jumper et al.'s^2^ AlphaFold2 model, which is capable of predicting accurate protein structures even without comparable structures for reference, demonstrated greater accuracy than other methods in CASP14, significantly advancing the field of protein structure prediction. Baek et al.^19^ developed the RoseTTAFold language model, a standalone model with an accuracy comparable to that of monomeric AlphaFold2 and AlphaFold2 multimers for complexes, which offers better computational scalability for proteins and complexes with more than 1000 residues. Overall, protein language models are increasingly favored by scholars because of their broader generalization capabilities and faster speeds. On the other hand, many scholars have treated proteins as textual sequence problems, extracting feature embeddings of protein sequences using word2vec. Heinzinger et al.^28^ utilized the SeqVec method, which effectively captures the biophysical properties of the language of life, and achieved significant improvements in predicting secondary structures and intrinsically disordered regions over standard encoding methods through simple neural network training, enhancing the performance of popular methods and even surpassing the best methods for certain proteins. Sharma et al.^29^ proposed a deep learning model called Deep-ABPpred, which combines bidirectional long short-term memory (BiLSTM) networks with word2vec to predict antimicrobial peptides (ABP) in protein sequences, demonstrating the practicality of Deep-ABPpred in identifying new antimicrobial peptides within protein sequences.

To address the distillation issues in protein secondary structure prediction, the knowledge distillation method is commonly employed to enhance model results and reduce model size. Wang et al.^30^ developed the PSSM-Distil framework, specifically for protein secondary structure prediction (PSSP) with low-quality PSSMs, using knowledge distillation and contrastive learning techniques to improve the prediction accuracy of low-quality PSSMs. Zhou et al.^23^ enhanced multiscale networks and further improved prediction accuracy using knowledge distillation techniques. Wang et al.^31^ applied knowledge distillation to their existing models for compression and acceleration while maintaining high predictive performance and enhancing computational efficiency. Given that AlphaFold2 is not particularly effective or efficient at removing nonhomologous sequences, Jing et al.^32^ employed AlphaFold2 to eliminate nonhomologous sequences. Additionally, scholars have attempted to apply knowledge distillation to datasets^33^.

In summary, scholars have integrated and optimized various models and explored the potential of language models and different feature extraction methods in understanding and generating protein sequences to improve protein secondary structure prediction. These studies demonstrate diverse advancements and continuously optimized approaches in the field of protein secondary structure prediction.

## Materials and methods

This section outlines the datasets and methodologies used in this study. Initially, six datasets were used to evaluate the model: five classical datasets (TS115, CB513, CASP13, CASP14 and CASP15) and a larger dataset from the PDB database. Feature extraction involves converting protein sequences into binary vector representations using one-hot encoding and considering the physicochemical properties of amino acids. The improved TCN model incorporates multiscale fusion and bidirectional operations to better capture amino acid sequence features. The BiLSTM model with a multi-head attention mechanism enhances the understanding of complex relationships in protein sequences. Knowledge distillation from the ProtT5 pretrained model is also employed to further improve the model's performance.

### Datasets

To evaluate the effectiveness of the model, three datasets, TS115, CB513, and 15,078 protein data points from 2018-06-06 to 2020, were used as training and test sets. The data in the PDB were selected from X-ray crystallography, with a resolution of at least 2.5 angstroms, no chain breaks, and fewer than 5 unknown amino acids. The CB513 dataset contains 513 protein sequences, a nonhomologous set created by Cuff and Barton, while the TS115 dataset contains 115 protein sequences.

In addition, to verify its effectiveness on public datasets, this paper uses 15,078 proteins from 2018-06-06 to 2020 obtained from the PDB database as training sets and protein sequences from free template modeling (FM) of the CASP13, CASP14, and CASP15 public datasets as test sets. The datasets of CASP13, CASP14, and CASP15 were obtained from the official website of the CASP competition (https://predictioncenter.org/), and their secondary structure sequences were predicted by DSSP. The selection of the CASP dataset in this paper refers to the definition of Chen et al.^34^, who used free template modeling data for CASP13 (11 sequences), CASP14 (9 sequences), and CASP15 (11 sequences) and excluded the large protein structures therein.

Generally, the secondary structure of proteins and peptides can be defined as 8 states, namely, H (α helix), G (3 10 helix), I (π helix), E (extended β strand), B (separated β strand), T (turn), S (bend) and others (C); in addition, many scholars usually merge (E, B) into E, (H, G, I) into E, and (C, S, T) into C, simplifying the above 8 states (Q_8_) into 3 states (Q_3_).

For TS115, CB513 and the data intercepted from the PDB (2018–2020) database, this paper randomly divides them 7:3 for training and testing, with the former used as the training set and the latter used as the test set; for verifying the CASP data, this paper uses the data intercepted from the PDB (2018–2020) database as the training set and the CASP data used as the test set.

### Feature extraction

Here, the model in this article uses the word2vec technique to process the protein primary structure sequence for feature extraction and word vector generation. For the input features of proteins, two key factors are considered in this article: the use of one-hot coding to represent the protein sequence and the consideration of the physicochemical properties of amino acids. Determination of amino acid physicochemical properties and one-hot coding provides direct, simple and effective methods for obtaining basic information about protein sequences. This coding approach can intuitively reflect the properties of amino acids and the composition of sequences, thus providing clear feature inputs for models. In contrast, other coding approaches usually require more computational resources and more complex data preprocessing.

First, this article uses one-hot coding to convert the amino acid sequence of a protein into a binary vector representation, where each amino acid corresponds to a unique binary vector. This approach can be used to effectively determine the presence or absence of amino acids in a protein sequence, providing important information for subsequent analysis. Second, the physicochemical properties of the amino acids used in this study were considered. This includes properties such as the polarity, charge, and size of the amino acid. By using these properties as additional features, the protein sequence can be characterized more comprehensively, thus providing additional information for further analysis.

In this article, this two-dimensional tensor is spliced using the concatenation operation, which retains complete information with more adaptability and flexibility than other splicing methods and is adaptable and flexible enough that it may be able to achieve better results for smaller amounts of data.

**Figure 1:**
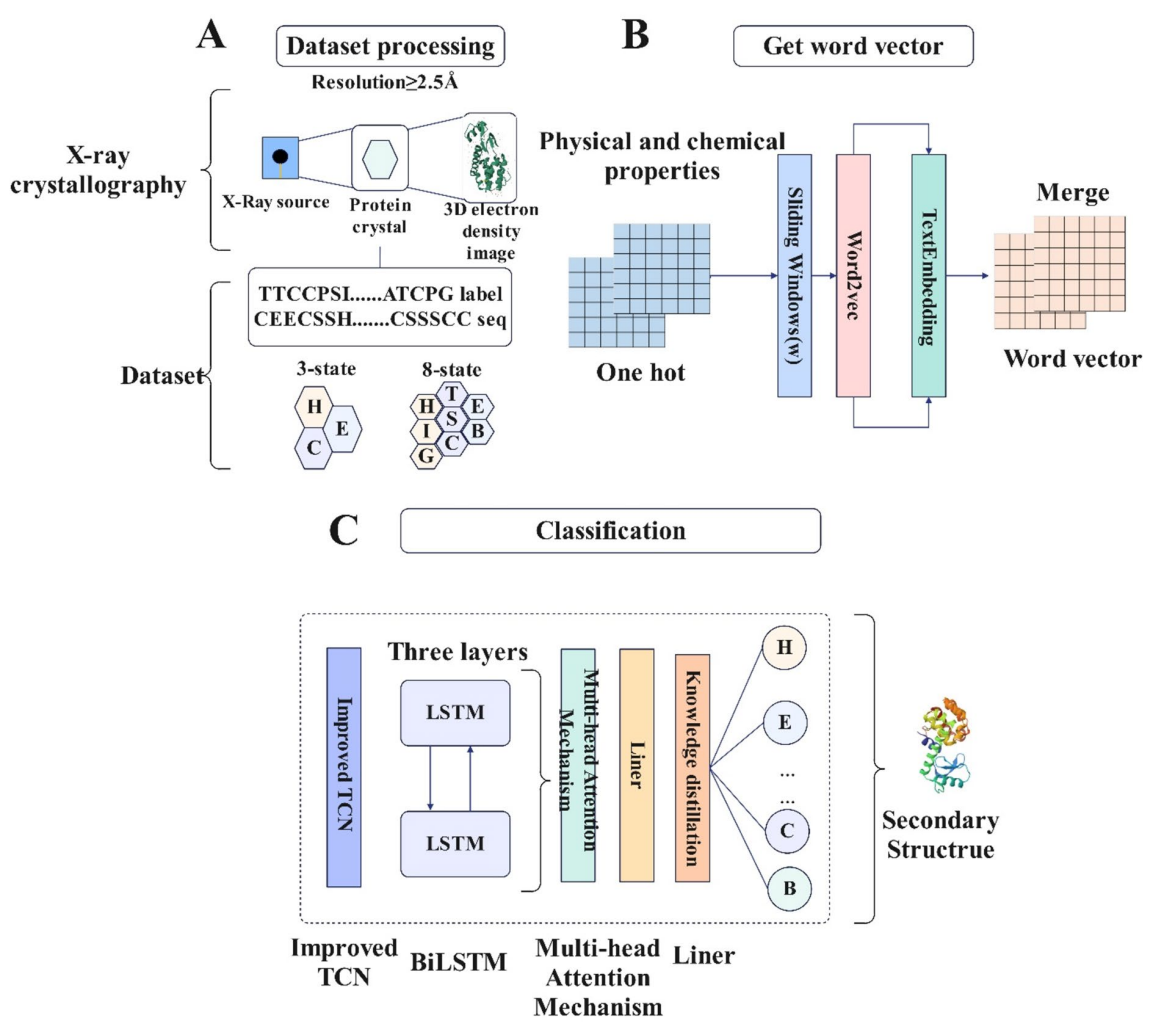
The workflow and framework.

Using a combination of one-hot encoding and the physicochemical properties of amino acids, input features for training word2vec embeddings can be generated to obtain a vector representation of the protein sequence. Finally, predictions are made using the model proposed in this article, and the complete flow of the article is shown in Fig. 1. Where, A: Dataset processing, including X-rays, produces a 3D electron density image of the protein structure and the sequence and labels the dataset contains; B: Get Word Vector, Transform protein sequences into comprehensive feature representations using physical and chemical properties, one-hot encoding, sliding window, Word2vec, and train embeddings; C: Predict protein secondary structure using a model combining improved TCN, BiLSTM, multi-head attention, a linear layer, and knowledge distillation from ProtT5-XL-UniRef. (The protein image is from the RCSB PDB database (https://www.rcsb.org/) Protein ID 1L58).

### Methods

The well-preprocessed data are first fed into the TextEmbedding layer to embed the word vector features, where a dropout layer equal to 0.2 is added to better output the features. To enable the network to extract localized features, the input features are segmented into individual short sequences using the sliding window technique and are subsequently fed into the improved TCN layer. Typically, the ResBlock module of the TCN includes two convolutional layers, which can effectively optimize the deep learning problem. To further enhance the TCN, this article adds the ResBlock module as three layers. Additionally, compared to the ordinary TCN layer, the improved TCN layer divides the model into forward and backward parts, each of which contains multiple ResBlocks with the same structure, to capture the different scales and directions of the text sequences, thereby improving the understanding of the text information.

In the BiLSTM model, three bidirectional LSTM layers with powerful analytical capabilities are used to extract the key global interactions in the protein sequences; in addition, two dropout layers are added to this model to ensure gradient stabilization during training. At the same time. A multi-head attention mechanism is added, which is more suitable for understanding complex relationships and patterns in protein sequences than traditional attention mechanisms.

**Figure 2:**
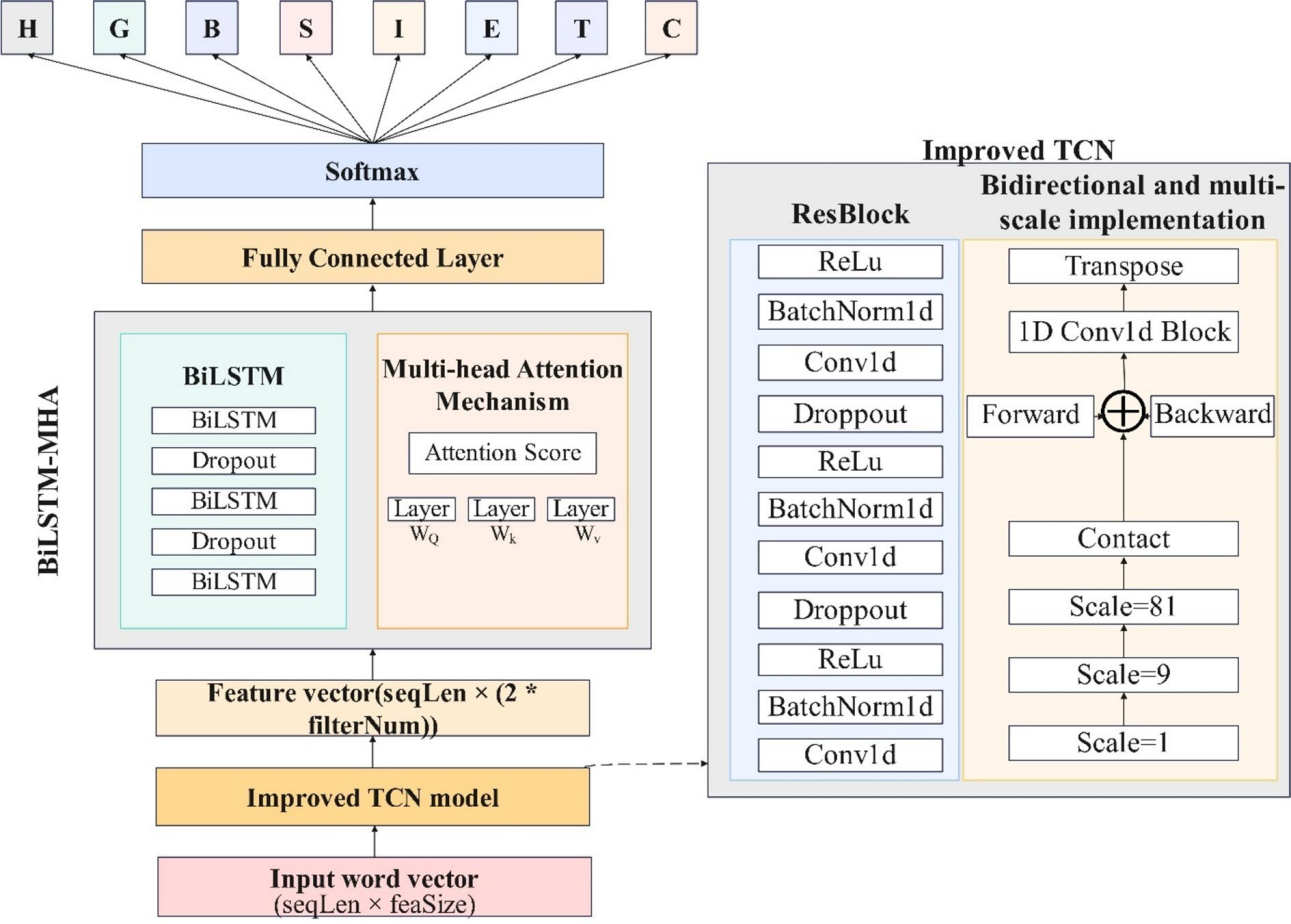
Protein secondary structure prediction model of this article.

In addition, to effectively learn more protein structure features, this model uses distillation learning. ProtT5-XL-UniRef, which is currently the most effective protein language model, was used as the teacher learning model in this article. Because ProtT5-XL-UniRef has more data and features, this study aimed to improve the effectiveness of this model. The specific model structure is shown in Fig. 2:

#### Improved TCN

In this article, a multiscale and bidirectional processing model is adopted because a single TCN can extract unidirectional features but does not make good use of spatial information or before-and-after relationships. According to previous work, there have been practices such as multiscale extraction of spatial features to improve TCNs in different fields^35^, and the use of bidirectional TCNs has also been considered; however, none of them can extract information comprehensively. Specifically, this article uses multiple Resblcok convolutional layers of different sizes and scales in the TCN model and uses the Concat method to fuse them. Each convolutional layer has a different receptive field. These convolutional layers are located in the feature extraction part of the TCN model and can capture local and global features in the protein sequence. By fusing these features of different scales, a more comprehensive feature representation can be obtained.

At the same time, for the model input to the multiscale TCN, this article performs bidirectional processing on the sequence, which means processing the data from front to back and from back to front at the same time to capture the bidirectional dependencies in the data. The model processes not only from the beginning to the end of the sequence (forward) but also from the end of the sequence to the beginning (backward). These bidirectional convolutional layers are also located in the feature extraction part of the TCN model to ensure that the model can simultaneously consider the dependencies between the front and back of the sequence. The information obtained from forward and backward processing is integrated, allowing the model to have a more comprehensive understanding of the relationships in the protein sequence.

- Multiscale TCN model

Multiscale protein sequence modeling is achieved by using convolution kernels of different scales. Specifically, for each scale, residual blocks containing multiple convolutional layers with convolutional kernels of different sizes are used; in this article, multiple scales are used separately to perform the cuts, and the outputs of each scale are fused together by the contour method; then, a 1 × 1 convolutional layer is used to keep the output dimensionality consistent.

First, the word vector of the word2vec preprocessed protein sequence obtained by using the mapping space that combines one-hot coding and the physicochemical properties of amino acids is denoted as X = {x_1_, x_2_…, x_L_}, where L represents the maximum length of the word vector of the preprocessed amino acid sequence of the protein, which is first inputted into the causal dilation convolution. Thus, for a given scale *s*, a convolution operation is performed on multiple scales, as described earlier, to obtain Eq. (1):

1 $$  o_{t,s} = \mathop \sum \limits_{i = 0}^{{k_{s} - 1}} f_{i,s} \cdot x_{{t - d_{s} \cdot i}} $$

Here, *k*_*s*_ is the convolutional kernel size for scale *s*, *f*_*i,s*_ are the corresponding convolutional kernel weights, and *d*_*s*_ is the expansion factor. Next, the multiscale outputs at time step *t* are merged using a 1 × 1 convolutional layer, as shown in Eq. (2) is shown:

2 $$ O_{t} = 1D{\text{Conv}}\left( {o_{t,1} ,o_{t,2} , \ldots ,o_{t,S} } \right) $$

Due to the dilated convolutional architecture of the TCN, the hidden layer in the middle of the network loses considerable important feature information. Therefore, this article further proposes a multiscale TCN model (MSTCN) to more comprehensively utilize residual features for classification. This approach can overcome the problem of feature information loss in the middle layer due to the dilated convolutional architecture in the standard TCN in the analysis of protein sequences and better preserve and utilize key information related to the structure and function of protein sequences.

To further optimize the multiscale TCN model in this article, bidirectional information capture is introduced to construct the new improved TCN model, and the specific working principle of the improved TCN model is shown in Fig. 2. Let $\overrightarrow {MSTCN}$ and $\overleftarrow {MSTCN}$ be the models of forward and reverse MSTCNs, respectively; the output expressions and computations of forward $\vec{Y}$ and reverse $\mathop{Y}\limits^{\leftarrow}$ are shown in *Eqs*. (3), (4):

3 $$ \vec{Y} = \overrightarrow {MSTCN} \left( X \right) $$

4 $$ \mathop{Y}\limits^{\leftarrow}  = \overleftarrow {MSTCN} \left( X \right) $$

First, the forward and reverse outputs are merged and subjected to a one-dimensional convolution process to obtain $Y = \vec{Y} \oplus \mathop{Y}\limits^{\leftarrow}$, while the weights and biases of the fully connected layers are applied to obtain *Z,* as shown in Eq. (5). where *W* and *b* are the weight matrices and bias terms of the fully connected layer, respectively.

5 $$ Z = W \cdot 1D{\text{Cov}}\left( Y \right) + b $$

Thus, the final output is as in Eq. (6)*,* and for moment *t,* as in Eqs. (7), (8)

6 $$ {\text{ Output }} = {\text{ BatchNormal }}\left( {f\left( Z \right)} \right) $$

7 $$ \hat{y}_{t} = \overrightarrow {MSTCN} \left( {x_{1} ,x_{2} , \ldots ,x_{t} } \right) \oplus \overleftarrow {MSTCN} \left( {x_{L} ,x_{L - 1} , \ldots ,x_{t} } \right) $$

8 $$ {\text{Output }}_{t} = {\text{ BatchNormal }}\left( {f\left( {W\left( {1D{\text{Cov}}\left( {\hat{y}_{t} } \right)} \right) + b} \right)} \right) $$

where $\oplus$  is the addition operation of the matrix, 1DCov is the 1 × 1 convolution operation of the residual block, BatchNormal is the pooled connectivity layer, softmax is the activation function *f* for classification, and Output is the final output of the BTCN. On a multiscale basis, the forward and backward information of each scale is fused, and the integrated multiscale feature representation is ultimately converted to the final output using a 1 × 1 convolutional layer. Finally, the model outputs a comprehensive feature map including detailed spatial and temporal information from multiple scales and two directions as the intermediate output of the improved TCN model.

For the TCN model, which employs multiscaling with a more robust range of causal extensions and therefore introduces simultaneous processing of forward and backward information, the model provides a more comprehensive understanding of the contextual information in the input sequences. This helps to capture the complex dependencies and contexts in the sequences; at the same time, bidirectional information capture helps to improve the generalization performance of the model, making it more applicable to different tasks and datasets.

#### BiLSTM-MHA

An LSTM network is a special type of recurrent neural network (RNN). Through its unique structural design and gating mechanism, LSTM is able to capture dependencies in data over longer time scales because protein secondary structure prediction involves identifying the structural type of each amino acid in the protein sequence, which is a sequence-to-sequence prediction problem. Therefore, the LSTM network can effectively capture the relationships between amino acids for prediction. This article adds a bidirectional operation on the basis of LSTM, and BiLSTM is able to process data from both the forward and backward directions of the sequence. This means that it can not only use the information before the current amino acid but also use the information after it for prediction. This is very important for protein secondary structure prediction because the structural characteristics of an amino acid may be affected by the amino acid before and after it.

To further enhance the model, this article integrates the multi-head attention mechanism with BiLSTM. The multi-head attention mechanism allows the model to jointly focus on the information of different representation subspaces of the encoder BiLSTM hidden state when decoding. The context vector obtained by the traditional attention mechanism focuses on a specific representation subspace of the input sequence, and this context vector is expected to reflect certain semantic aspects in the input. However, considering that protein sequences usually contain multiple related semantic spaces, especially when dealing with long sequences, the multi-head attention mechanism can enable the protein secondary structure prediction model to capture richer and more detailed features in different subspaces, which helps to predict protein secondary structure more accurately.

Therefore, this article is the first to use an improved TCN to extract multiscale features from protein sequences. The improved TCN captures local and global dependencies in the sequence through its expansion and residual connection, and its output is input into the BiLSTM-MHA model as a rich feature representation to output the prediction probability.

- BiLSTM model

A standard LSTM at time *t* mainly consists of a forget gate *f*_*t*_, a reset gate *i*_*t*_ and an output gate *o*_*t*_; the LSTM cell-specific equations are shown in Eqs. (9), (14):

9 $$ i_{t} = \sigma \left( {X_{t} W_{Hi} + h_{t - 1} W_{hi} + b_{i} } \right) $$

10 $$ f_{t} = \sigma \left( {X_{t} W_{Hf} + h_{t - 1} W_{hf} + b_{f} } \right) $$

11 $$ o_{t} = \sigma \left( {X_{t} W_{Ho} + h_{t - 1} W_{ho} + b_{o} } \right) $$

12 $$ \tilde{c}_{t} = {\text{tanh}}\left( {X_{t} W_{Hc} + h_{t - 1} W_{hc} + b_{c} } \right) $$

13 $$ c_{t} = f_{t} c_{t - 1} + i_{t} \mathop c\limits^{`}_{t} $$

14 $$ h_{t} = {\text{tanh}}\left( {c_{t} } \right)o_{t} $$

At time step $t$, the input of the LSTM is $H_{t}$, which represents the input sequence after the TCN extracts features. Among them, $H = \left\{ {H_{1} ,H_{2} , \ldots ,H_{L} } \right\}$. *L* is the length of the sequence, where $W_{Hi} ,W_{Hf} ,W_{Ho}$ and $W_{Hc}$ are the weight matrices of each layer connected to the input vector $H_{t}$; $W_{hi} ,W_{hf} ,W_{ho}$ and $W_{hc}$ are the weight matrices of each layer connected to the previous hidden state $h_{t - 1}$; and $b_{i} ,b_{f} ,b_{o}$ and $b_{c}$ are the bias parameters. The sequence at time *t* passes through the LSTM network and outputs the hidden state $h_{t}$.

**Figure 3:**
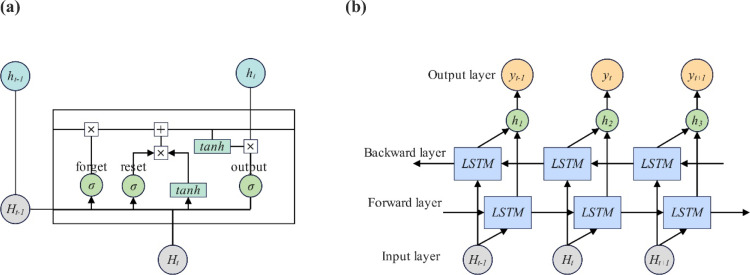
BiLSTM Model: (**a**) LSTM cell; (**b**) BiLSTM network.

The combined output of BiLSTM at time step *t* is the result of concatenating the forward and backward passes through the LSTM network. The specific output is shown in Eq. (20):

15 $$ \overleftrightarrow {h_{t} } = {\text{Concat}}\left( {\overrightarrow {{{\text{LSTM}}\left( {H_{t} } \right)}} + \overleftarrow {{{\text{LSTM}}\left( {H_{t} } \right)}} } \right) $$

where $\overrightarrow {{{\text{LSTM}}\left( {H_{t} } \right)}}$ and $\overleftarrow {{{\text{LSTM}}\left( {H_{t} } \right)}}$ represent the outputs of the LSTM of the forward and reverse sequences, respectively, and $\overleftrightarrow {h_{t} }$ represents the output of the BiLSTM model at time *t*. The specific representation is shown in Fig. 3 BiLSTM Model:

(b) Multi-head attention mechanism

The attention mechanism can selectively focus on features that are more relevant to the task and suppress the influence of useless features, thereby improving the performance of the model. For a given *q* and *X*, the probability of selecting the *i* input information $\alpha_{i}$ is given by Eq. (16):

16 $$ \alpha_{i} = p\left( {z = i{\mid }H,q} \right) = {\text{softmax}}\left( {s\left( {x_{i} ,q} \right)} \right) = \frac{{e^{{s\left( {x,q} \right)}} }}{{\mathop \sum \nolimits_{j = 1}^{N} e^{{s\left( {x,q} \right)}} }} $$

Here, $z$ represents the index position, the dimensions of the input information $H = \left[ {\overleftrightarrow {h_{1} },\overleftrightarrow {h_{2} }, \ldots ,\overleftrightarrow {h_{N} }} \right]$ are $N,q$ is the query matrix, and $s\left( {x_{i} ,q} \right)$ is the attention scoring function. The corresponding formula is shown in Eq. (17):

17 $$ s\left( {x_{i} ,q} \right) = \frac{{x_{i}^{T} q}}{\sqrt d } $$

Here, *d* is the dimension of the input information. The scaled dot product attention function used is shown in Eq. (18):

18 $$ {\text{Attention}}\left( {Q,K,V} \right) = {\text{softmax}}\left( {\frac{{QK^{T} }}{{\sqrt {d_{k} } }}} \right)V $$

**Figure 4:**
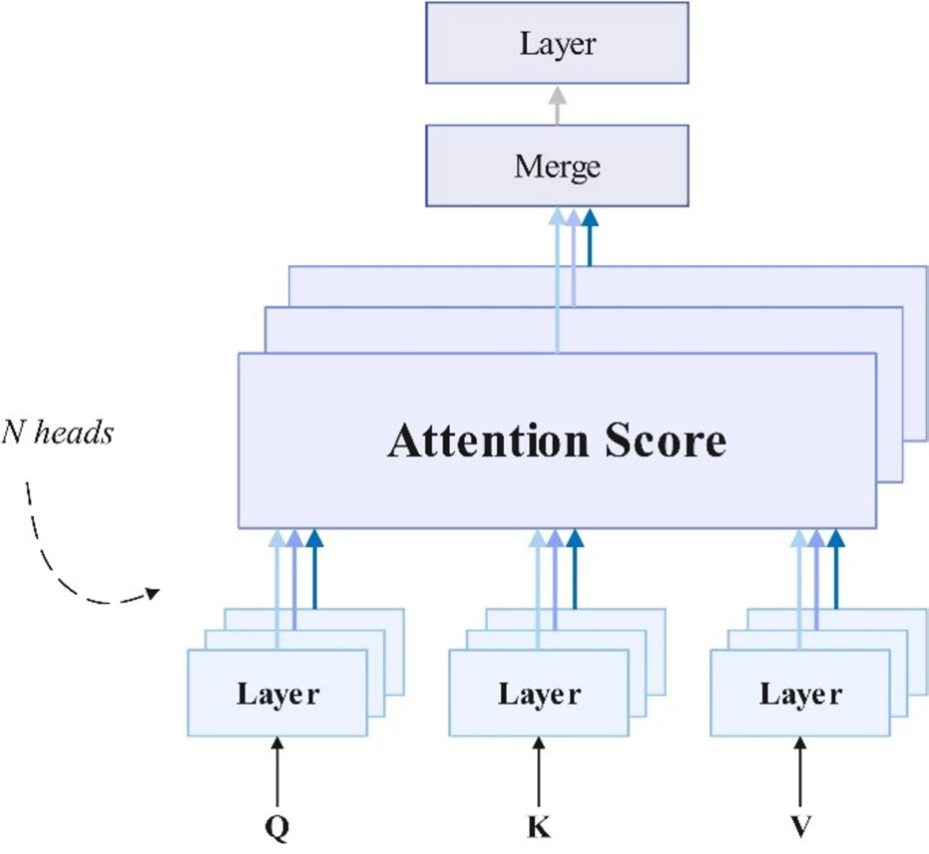
Multi-head attention mechanism.

*Q* is the query matrix, *K* is the keyword matrix, and *V* is the numerical matrix of the keywords. The multi-head attention mechanism is composed of multiple self-attention mechanism structures and is used to process the same feature information at the same time. Its output is the splicing of multiple self-attention mechanisms. In this article, three attention mechanisms are used for splicing. As shown in Fig. 4. This structure can better capture the dependencies between different features and further improve the performance of the model. Its expression is Eq. (19):

19 $$ {\text{MultiHead}}\;\left( {Q,K,V} \right) = {\text{Concat}}\left( {{\text{head}}_{1} , \ldots ,{\text{head}}_{n} } \right)W^{o} $$

where ${\text{head}}_{i} = Attention\left( {QW_{i}^{Q} ,KW_{i}^{K} ,VW_{i}^{V} } \right),W_{i}^{Q} ,W_{i}^{K} ,W_{i}^{V}$ is the mapping matrix weight, and $W^{o}$ is the output weight matrix.

(c) BiLSTM-MHA model

Finally, the BiLSTM output is concatenated with the multi-head attention mechanism, and the final result is processed by a 1 × 1 convolutional layer to maintain dimensionality consistency and generate a feature map ${Z}_{t}$ suitable for classification. The calculation method is shown in Eq. (20):

20 $$ Z_{t} = {\text{1DCov}}\left( {{\text{Concat}}\left( {\overleftrightarrow {h_{t} },{\text{Multi - head}}\left( {Q,K,V} \right)} \right)} \right) $$

Next, as shown in Eq. (21), the output feature map $$Z $$ passes through a fully connected layer with a softmax activation function to generate the final category probability:

21 $$ P_{t} = {\text{softmax}}\left( {WZ_{t} + b} \right) $$

Here, *W* and *b* are the weights and biases of the fully connected layer, respectively, and $P_{t}$ represents the probability distribution at time step $t$. Therefore, the output of the BiLSTM model is a series of probability distributions $P = \left\{ {P_{1} ,P_{2} , \ldots ,P_{L} } \right\}$, which can be used for the final classification.

#### Knowledge distillation

To effectively learn the complex features and computational power of the protein language model, this article uses knowledge distillation for this purpose. The purpose of knowledge distillation is to extract knowledge from knowledge to transform the cumbersome teacher model into a lightweight student model so that the students can work with data similar to or more harpy than the teacher model. Knowledge distillation guides the training of the student model by providing softened softmax labels through the teacher model. Given the output of the teacher model and the students’ *Z*_*T*_ and *Z*_*S*_. The student model represents the improved TCN-BiLSTM-MHA proposed above, and the teacher model is ProtT5-XL-UniRef. They are tested on various datasets proposed in this article, and the corresponding outputs are obtained.

**Figure 5:**
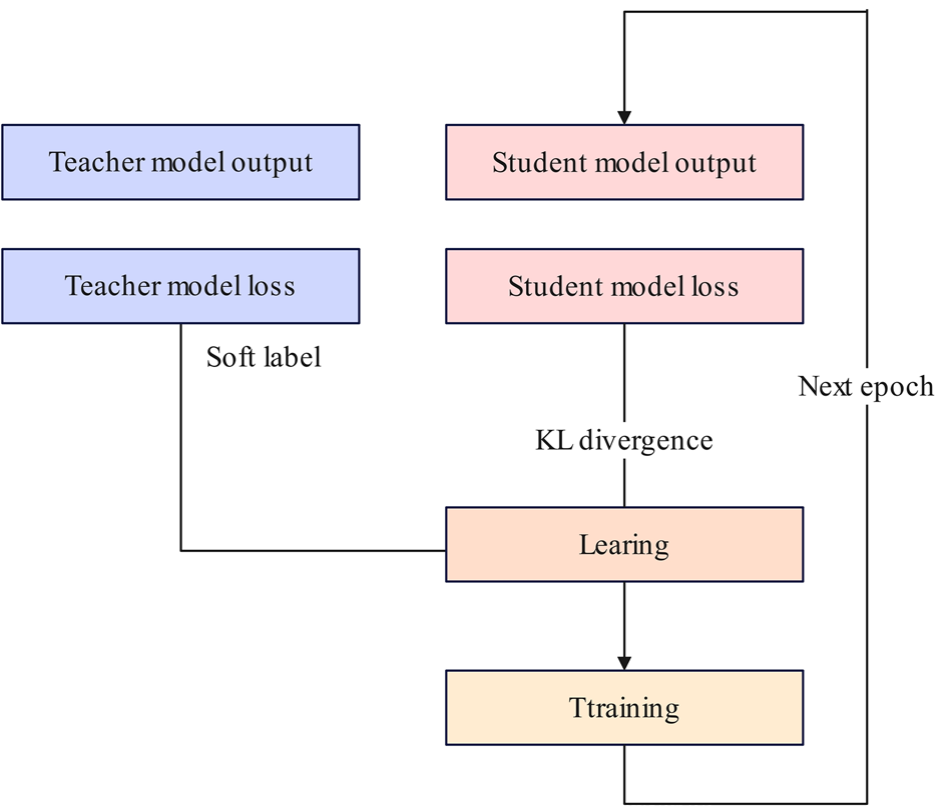
Knowledge distillation method.

In the model, the distillation losses are calculated as Eq. (22):

22 $$ {\mathcal{L}}_{KD} = {\mathcal{H}}\left( {\sigma_{S} \left( {Z_{S} ;\rho } \right),\sigma_{T} \left( {Z_{T} ;\rho } \right)} \right) $$

where H is the negative cross-entropy loss or KL dispersion and $\sigma$ denotes the softmax of the $\rho$ temperature. When $\rho$ is greater than the output probability, the output probability is smoother, more information is carried out, the influence of negative labels is relatively amplified, and model training pays more attention to negative labels. In this knowledge distillation, this article selects the KL scatter distillation. The specific flow chart is shown in Fig. 5:

## Experiment

### Evaluation metrics

In this article, three primary evaluation metrics were used to assess the performance of our protein secondary structure prediction models: the segment overlap measure 99 (SOV99), ACC (accuracy), and MiAUC (microaveraged area under the curve). Each of these metrics provides a different perspective on the model's performance, offering a comprehensive evaluation.

- SOV99

The SOV99^36^ is a widely used metric for evaluating the accuracy of protein secondary structure predictions. SOV99 is a specific variant that considers the overlap between predicted and actual segments of secondary structure elements. The main differences between SOV and SOV99 are the normalization procedures and the definition of the degree of variation in the fragment boundaries. SOV99 improves upon the original SOV by normalizing the values to be calculated based on the fragments assigned to the reference, lowering the prediction score to accurately reflect the paired nature of the segment comparison so that the score ranges from 0 to 100%, facilitating direct comparison with other evaluation metrics. In addition, SOV99 places further restrictions on the degree of variation of fragment boundaries so that the variation range cannot exceed half of that of the shorter fragment, thereby more strictly identifying similar and dissimilar fragment distributions. These improvements improve the accuracy and usefulness of SOV99 in protein secondary structure prediction.

The 8-state secondary structures are H, G, I, E, B, S, T, and C, and the 3-state secondary structures are H, E, and C. This article uses ${\text{s}}_{1}$ and ${\text{s}}_{2}$ to denote segments of the secondary structure in the conformational state $i$. The segments ${\text{s}}_{1}$ and ${\text{s}}_{2}$ correspond to the two secondary structure assignments being compared. The first assignment is considered a reference and is typically based on experiments; the second assignment is the one being evaluated. The two assignments are further referred to as “observed” and “predicted,”, respectively. ${\text{s}}_{1} ,{\text{ s}}_{2}$ denotes a pair of overlapping segments, $S\left( i \right)$ denotes the set of all the overlapping pairs of segments $\left( {{\text{s}}_{1} ,{\text{ s}}_{2} } \right)$ in state $i$, and $S^{\prime}\left( i \right)$ denotes the set of all segments $s_{1}$ for which there is no overlapping segment ${\text{s}}_{2}$ in state $i$, as shown in Eqs. (23), (24):

23 $$ S\left( i \right) = \left\{ {\left( {s_{1} ,s_{2} } \right):s_{1} \cap s_{2} \ne \emptyset } \right.{, }s_{1} \;{\text{and}} \;s_{2} \;{\text{are}}\;{\text{ both}}\;{\text{ in }}\;{\text{conf}}\left. {{\text{ormational }}\;{\text{state\,i}}} \right\} $$

24 $$ S^{\prime}\left( i \right) = \left\{ {s_{1} :\forall s_{2} ,s_{1} \cap s_{2} = \emptyset } \right.{, }\;s_{1} \;{\text{and}}\;s_{2} \;{\text{are}}\;{\text{ both}}\;{\text{ in }}\;{\text{conformational}}\;{\text{ stateij}} $$

Sov is a metric based on the ratio of overlapping segments, which is defined as:

25 $$  SOV = 100 \times \left[ {\frac{1}{N}\mathop \sum \limits_{i} \mathop \sum \limits_{S\left( i \right)} \frac{{{\text{minov}}\left( {s_{1} ,s_{2} } \right) + \delta \left( {s_{1} ,s_{2} } \right)}}{{{\text{maxov}}\left( {s_{1} ,s_{2} } \right)}} \times {\text{len}}\left( {s_{1} } \right)} \right] $$

As shown in Eq. (25), len $\left( {{\text{s}}_{1} } \right)$ is the number of residues in segment $s_{1}$, minov $\left( {s_{1} ,s_{2} } \right)$ is the length of the actual overlap of $s_{1}$ and ${\text{s}}_{2}$,${\text{maxov}}\left( {{\text{s}}_{1} ,{\text{ s}}_{2} } \right)$ is the total extent for which either of the segments ${\text{s}}_{1}$ and ${\text{s}}_{2}$ has a residue in state $i$, and $\delta \left( {{\text{s}}_{1} ,{\text{ s}}_{2} } \right)$ is defined as Eq. (26):

26 $$ \delta \left( {{\text{s}}_{1} ,{\text{ s}}_{2} } \right) = {\text{min}}\left\{ {\begin{array}{*{20}c} {\left( {{\text{maxov}}\left( {{\text{s}}_{1} ,{\text{ s}}_{2} } \right) - {\text{minov}}\left( {{\text{s}}_{1} ,{\text{ s}}_{2} } \right)} \right)} \\ {minov\left( {{\text{s}}_{1} ,{\text{ s}}_{2} } \right)} \\ {int\left( {{\text{len}}\left( {{\text{s}}_{1} } \right)/2} \right);int\left( {{\text{len}}\left( {{\text{s}}_{2} } \right)/2} \right)} \\ \end{array} } \right. $$

where ${\text{min}}\left[ {x1;x2;x3; \ldots ;xn} \right]$ is the minimum of $n$ integers. The normalization value $N{ }$ is defined as Eq. (27):

27 $$  N\left( i \right) = \mathop \sum \limits_{i} \left( {\mathop \sum \limits_{S\left( i \right)} {\text{len}}\left( {s_{1} } \right) + \mathop \sum \limits_{S\left( i \right)} {\text{len}}\left( {s_{1} } \right)} \right) $$

SOV99 takes into account the continuity of the predicted segments, not just the individual residues. This is crucial for protein structure predictions because it emphasizes the correct prediction of entire secondary structure segments rather than just individual amino acids. A higher SOV99 indicates that the predicted segments align well with the true segments in terms of their boundaries and lengths.

(b) ACC

Accuracy is a basic metric that measures the proportion of correctly predicted secondary structure elements (such as alpha-helices, beta-strands, and coils) out of the total number of elements. In this article, accuracy (Q_8_) and accuracy (Q_3_) are used to measure the goodness of fit of the model. The ACC (Q3) and ACC (Q8) are the ratios of the number of correctly numbered residues predicted to be the number of all the residues S, which are defined as Eqs. (28), (29):

28 $$ {\text{ACC}}\left( {{\text{Q}}_{3} } \right) = \frac{{S_{C} + S_{E} + S_{H} }}{S} \times 100 $$

29 $$ {\text{ACC}}\left( {{\text{Q}}_{8} } \right) = \frac{{S_{H} + S_{G} + S_{I} + S_{E} + S_{B} + S_{C} + S_{T} + S_{S} }}{S} \times 100 $$

where ${\text{S}}_{i} \left( {i\, \in \,\left\{ {{\text{H}},{\text{E}},{\text{C}}} \right\}{\text{or}}\left\{ {{\text{H}},{\text{G}},{\text{I}},{\text{E}},{\text{BC}},{\text{T}},{\text{S}}} \right\}} \right)$ denotes the correct number of individual types *i* predicted. The accuracy provides an overall measure of how well the model predicts the correct secondary structure elements. It is a straightforward and intuitive metric, but it does not distinguish between different types of errors or account for the balance between classes.

(c) MiAUC

The microaveraged area under the curve (MiAUC) is an important indicator for evaluating the performance of multiclassification models. The area under the ROC curve (AUC) is calculated based on microaveraging, which is particularly useful for dealing with multiclass classification problems. The MiAUC can provide a more comprehensive model performance evaluation by integrating the prediction results of all categories to calculate the overall AUC value. For each class k, the true positive rate and false positive rate are calculated as shown in Eq. (30):

30 $$ \begin{aligned} TPR_{k} & = \frac{{TP_{k} }}{{TP_{k} + FN_{k} }} \\ FPR_{k} & = \frac{{FP_{k} }}{{FP_{k} + TN_{k} }} \\ \end{aligned} $$

Next, microaveraging is used to calculate the overall TPR and FPR. The true positives, false positives, true negatives, and false negatives of all categories are summed, and then the microaveraged TPR and FPR are calculated, as shown in Eq. (31):

31 $$ \begin{aligned} TPR_{{\text{micro }}} & = \frac{{\mathop \sum \nolimits_{k} TP_{k} }}{{\mathop \sum \nolimits_{k} \left( {TP_{k} + FN_{k} } \right)}} \\ FPR_{{\text{micro }}} & = \frac{{\mathop \sum \nolimits_{k} FP_{k} }}{{\mathop \sum \nolimits_{k} \left( {FP_{k} + TN_{k} } \right)}} \\ \end{aligned} $$

The ROC curve is plotted based on the microaveraged TPR and FPR values, as shown in Eq. (32). The area under the microaveraged ROC curve was calculated using the numerical integration method to obtain the MiAUC:

32 $$ {\text{MiAUC }} = \mathop \smallint \limits_{0}^{1} TPR_{{\text{micro }}} \left( {FPR_{{\text{micro }}} } \right)d\left( {FPR_{{\text{micro }}} } \right) $$

The MiAUC is an important and commonly used indicator for comprehensively evaluating the performance of classification models, especially when dealing with imbalanced datasets.

### Experimental results and discussion

To understand the validity of the model results in this article, the experiments in this article were performed together on six datasets: TS115, CB513, CASP13, CASP14, CASP15 and PDB (2018–2020). The final model proposed in this article is the distillation-improved TCN-BiLSTM-MHA model mentioned in section “Methods”. Moreover, to verify the validity of the structure and combinations, this article uses multiple modeling approaches for comparison. The results of the models on the TS115, CB513 and PDB (2018–2020) datasets are shown in Table 2.

**Table 2:** Comparative three-state performance results of various models on the TS115, CB513, and PDB (2018–2020) datasets.

Models	Ts115			CB513			PDB (2018–2020)	
	ACC (Q_3_)	MIAUC (Q_3_)	SOV99 (Q_3_)	ACC (Q_3_)	MIAUC (Q_3_)	SOV99 (Q_3_)	ACC (Q_3_)	MIAUC (Q_3_)
BiLSTM	87.3%	98.3%	34.1%	82.2%	97.0%	28.5%	*96.8%*	*99.8%*
BiLSTM-MHA	90.6%	98.4%	27.1%	88.1%	97.1%	28.6%	96.4%	**99.9%**
Improved TCN-BiLSTM- MHA	89.7%	99.0%	30.2%	88.0%	98.5%	32.6%	*96.8%*	**99.9%**
TCN-GRU	90.9%	**99.3%**	35.4%	87.8%	98.5%	28.8%	95.8%	**99.9%**
BiTCN-BiLSTM-MHA	87.9%	99.1%	30.7%	88.1%	98.1%	30.4%	**97.0%**	**99.9%**
Distillation- TCN-GRU	90.7%	*99.2%*	50.0%	89.1%	*98.7%*	44.2%	96.2%	**99.9%**
Distillation- BiTCN-BiLSTM-MHA	*91.2%*	**99.3%**	**53.3%**	**90.4%**	**98.8%**	**53.4%**	96.6%	**99.9%**
Distillation-Improved TCN-BiLSTM- MHA	**91.3%**	*99.2%*	*38.7%*	*90.3%*	98.5%	*49.5%*	*96.8%*	**99.9%**

For the TS115 and CB513 data, the ACC (Q_8_ and Q_3_), MiAUC (Q_8_ and Q_3_) and SOV99 (Q_3_) data were used. Due to the large amount of PDB (2018–2020) data, it was used as a training set to verify and compare the results of CASP13, CASP14, and CASP15, and ACC (Q_8_ and Q_3_), MiAUC (Q_8_ and Q_3_) and SOV99 (Q_3_) data were collected. The bold text indicates the best performance, and the italics text indicates the second-best performance. The results of all the tables in this section are expressed as follows.

- BiLSTM

Compared with the final model of this article, only BiLSTM is used as the prediction model, which is also the baseline model of this article. Capturing dependencies in both the forward and backward directions to improve contextual understanding in sequence prediction.

(b) BiLSTM-MHA

Compared with (a), a multi-head attention mechanism is added to the BiLSTM model, allowing the model to focus on different parts of the sequence at the same time, thereby performing more detailed feature extraction.

(c) Improved TCN-BiLSTM-MHA

Using the improved TCN model proposed in section “Improved TCN” of this article combined with BiLSTM and a multi-head attention mechanism, compared with the final model of this article, this model does not adopt knowledge distillation.

(d) TCN-GRU

This model combines the TCN model mentioned above with the GRU. Unlike the final model, only the basic TCN is used as the feature extraction model, and the GRU is used as the prediction model.

(e) BiTCN-BiLSTM-MHA

The bidirectional TCN and BiLSTM are combined with a multi-head attention mechanism. Compared with the final model in this article, its feature extraction model only uses a bidirectional TCN instead of an improved TCN and does not adopt a knowledge distillation method.

(f) Distillation-TCN-GRU

Compared with (d), knowledge distillation technology is used in the TCN-GRU model.

(g) Distillation-BiTCN-BiLSTM-MHA

Compared with (e), knowledge distillation technology is used on the BiTCN-BiLSTM-MHA model.

Table 2 shows that the distilled model generally works better than the model without distillation; at the same time, as the structure of the model in this article becomes more complex, the model works better, which on average will be 1–2% better than the structure of the previous layer; at the same time, all of the models will be better than the baseline.

For the three-state structure data, distillation had good results on both TS115 and CB513, suggesting that distillation will have some beneficial effect on three-state structure data on a certain small dataset. However, by comparison, the enhancement in the PDB (2018–2020) dataset may not be obvious, but the effect on the PDB (2018–2020) dataset is consistently better. Therefore, this article speculates that the first reason may be that the PDB (2018–2020) dataset has already obtained better results on the undistilled modeling method, so there is not much room for improvement; the second reason may be because the PDB (2018–2020) dataset is larger than CB513 and TS115 and may not obtain the same good results on the corresponding small datasets for the large datasets. The eight-state prediction results are shown in Table 3:

**Table 3:** Comparative three-state performance results of various models on the TS115, CB513, and PDB (2018–2020) datasets.

Models	Ts115		CB513		PDB (2018–2020)	
	ACC (Q_8_)	MiAUC (Q_8_)	ACC (Q_8_)	MiAUC (Q_8_)	ACC (Q_8_)	MiAUC (Q_8_)
BiLSTM	83.4%	99.0%	77.0%	98.5%	*95.2%*	*99.8%*
BiLSTM-MHA	86.0%	98.9%	83.3%	97.9%	**95.3%**	**99.9%**
Improved TCN-BiLSTM- MHA	84.4%	**99.2%**	83.3%	*98.7%*	**95.3%**	**99.9%**
TCN-GRU	86.3%	**99.2%**	84.3%	*98.7%*	94.4%	**99.9%**
BiTCN-BiLSTM-MHA	84.3%	**99.2%**	82.4%	*98.7%*	95.0%	**99.9%**
Distillation- TCN-GRU	*87.9%*	**99.2%**	84.8%	98.6%	95.0%	**99.9%**
Distillation- BiTCN-BiLSTM-MHA	**87.8%**	*99.1%*	**85.7%**	98.6%	95.1%	**99.9%**
Distillation-Improved TCN-BiLSTM- MHA	87.2%	*99.1%*	*84.9%*	**98.8%**	**95.3%**	**99.9%**

According to the eight-state structure data, distillation had a good effect on TS115 and a significantly more pronounced effect on CB513 according to the best model presented in this article. For the predicted eight-state structure, the smaller the dataset is, the more effective the distillation may be for this enhancement.

Compared with BiLSTM, the final model in this article has the following maximum improvements: an 8.1% improvement in ACC (Q_3_) on the CB513 dataset, a 1.0% improvement in MiAUC (Q_3_) on the TS115 dataset, and a 25.9% improvement in SOV99 (Q_3_) on the TS115 dataset for three-state structure data. For the eight-state structure data, the final model achieves a 7.9% improvement in ACC (Q_8_) on the CB513 dataset and a 1.0% improvement in MiAUC (Q_8_) on the TS115 dataset. These comparisons highlight the significant enhancements achieved by the final model across different datasets and metrics.

The results of using the extracted PDB (2018–2020) data as the training set and the three-state structure data of CASP13, CASP14, and CASP15 as the test set are shown in Table 4.

**Table 4:** Performance comparison of various models on the CASP13, CASP14, and CASP15 datasets.

Models	CASP13			CASP14			CASP15		
	ACC (Q_3_)	MiAUC (Q_3_)	SOV99 (Q_3_)	ACC (Q_3_)	MiAUC (Q_3_)	SOV99 (Q_3_)	ACC (Q_3_)	MiAUC (Q_3_)	SOV99 (Q_3_)
BiLSTM	68.1%	92.0%	29.6%	57.2%	86.1%	24.6%	57.1%	*86.4%*	**49.1%**
BiLSTM-MHA	67.6%	91.7%	29.1%	**58.2%**	*86.3%*	28.9%	*57.7%*	86.1%	*44.5%*
Improved TCN-BiLSTM- MHA	67.5%	91.7%	29.1%	56.8%	85.9%	43.9%	56.1%	85.9%	44.1%
TCN-GRU	67.3%	91.7%	25.5%	*57.7%*	86.1%	28.1%	56.4%	*86.4%*	42.4%
BiTCN-BiLSTM-MHA	66.7%	91.6%	33.4%	57.0%	85.9%	25.1%	56.5%	**86.5%**	43.2%
Distillation- TCN-GRU	67.8%	92.1%	*47.8%*	57.5%	**86.4%**	**47.4%**	57.1%	86.1%	36.4%
Distillation- BiTCN-BiLSTM-MHA	*68.2%*	*92.4%*	**51.4%**	56.4%	86.1%	*45.7%*	56.7%	86.2%	40.9%
Distillation-Improved TCN-BiLSTM- MHA	**69.2%**	**92.8%**	46.7%	56.7%	86.1%	43.4%	**58.4%**	85.8%	38.1%

Table 4 shows that the performance of the distillation-improved TCN-BiLSTM-MHA model on different datasets is generally better than that of the BiLSTM model. On the CASP13 dataset, the distillation-improved TCN-BiLSTM-MHA model improved the ACC (Q3) by 1.1%, the MiAUC (Q3) by 0.8%, and the SOV99 (Q3) by 17.1%. On the CASP14 dataset, the SOV99 (Q3) improved by 18.8%. On the CASP15 dataset, the ACC (Q3) was 1.3%.

#### Ablation study

**Figure 6:**
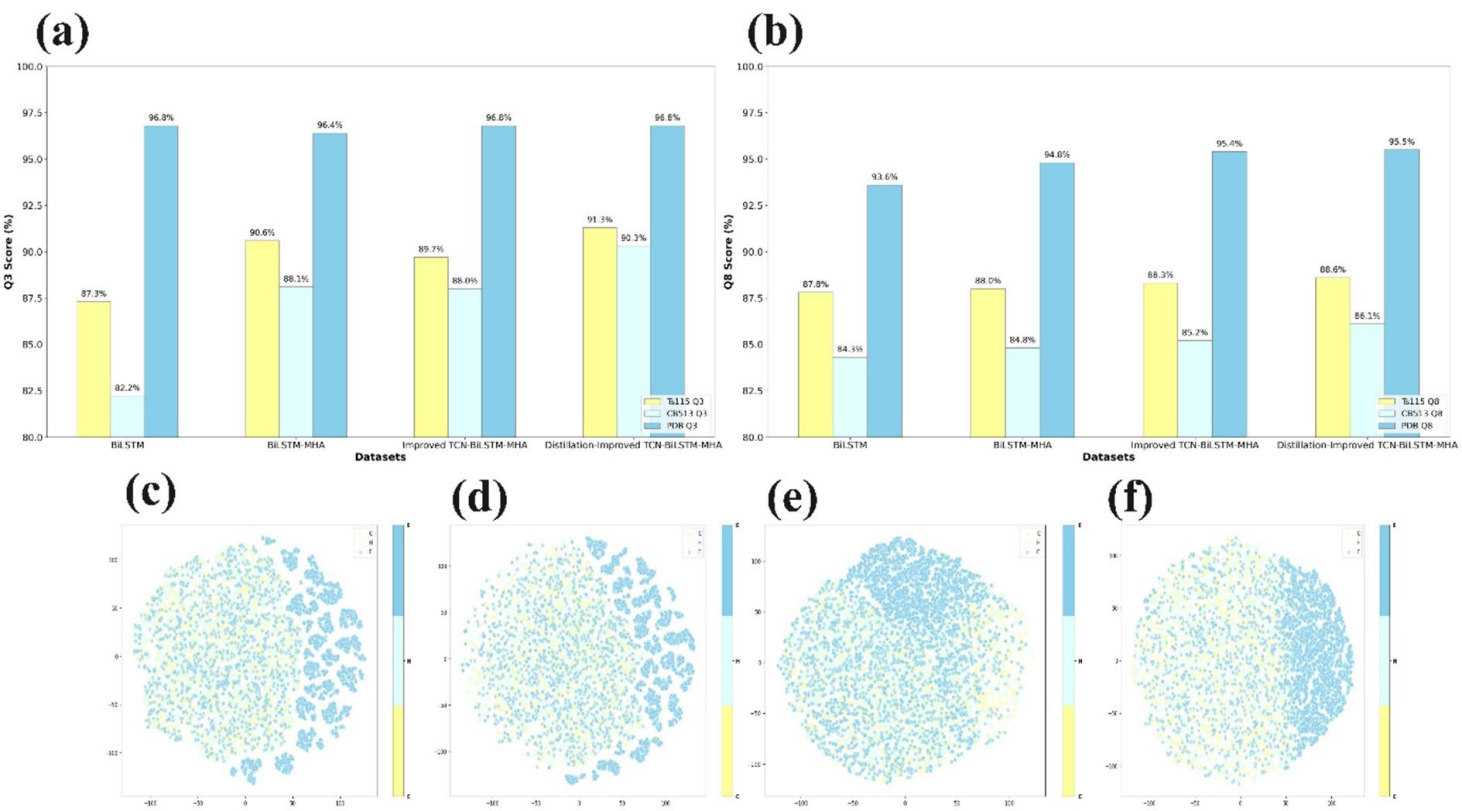
Ablation study: (**a**) comparison of ablation study results of different datasets on three-state structure; (**b**) comparison of ablation study results of different datasets on eight-state structure; (**c**–**f**) visualization of t-sne dimensionality reduction of BiLSTM, BiLSTM-MHA, improved TCN-BiLSTM-MHA and distillation-improved TCN-BiLSTM-MHA learned embedding features.

To further study the effectiveness of the model, relevant ablation experiments were performed, as shown in Fig. 6a, b, which show the performances of BiLSTM, BiLSTM-MHA, the improved TCN-BiLSTM-MHA and the distillation-improved TCN-BiLSTM-MHA on different datasets of eight-state and three-state structures of proteins. According to the ablation experimental study, each added module improved the model performance, indicating the validity of the model structure.

Moreover, to better understand the complementarity of each functional module, taking the TS115 dataset as an example, the embedded representations learned by these four models are visualized by t-distributed stochastic neighbor embedding (t-SNE) after dimensionality reduction, as shown in Fig. 6c–f:

In this article, it was found that both eight-state and three-state structures had better results on different datasets as the complexity of the model increased; on the other hand, the results of the t-sne dimensionality reduction visualization showed that the BiLSTM model alone could not extract the features very well, and as the model's complexity increased, the features of the protein’s amino acid sequences could be extracted in a better way to obtain a more effective modeling result.

#### Comparison with other methods

- Comparison with advanced algorithms

To better analyze the effectiveness of the proposed model in this article, the proposed model is compared with several state-of-the-art algorithms. Next, this article will briefly introduce the models in this chapter. CNN-LSTM and the Transformer are introduced in section “Advanced algorithms”.

MLPRNN^37^: The model consists of two multilayer perceptrons (MLPs) and a two-layer stacked bidirectional gated recurrent unit (BGRU). MLPRNN uses two types of input features: position-specific score matrix (PSSM) and hidden Markov model (HMM) features. These features are input into the first MLP block, which expands the input dimension from 41 to 512. Then, these expanded features are input into the BiGRU block to capture the long-range dependencies in the sequence. Finally, the output of the BiGRU passes through an MLP block again, which reduces the number of dimensions to 9 and is predicted through the Softmax layer.

GAN-BiRNN: The application of a GAN to protein secondary structures was proposed by Jin et al.^38^. The generator captures the complex features of protein sequences by combining one-dimensional convolution and multiscale convolution. The input features are the one-dimensional encoding and PSSM (position-specific score matrix) of the protein sequence, and the discriminator is used to determine whether the input secondary structure data are real or generated. The input is a combination of the output features of the generator and the real secondary structure data. This article adopts the GAN model and uses three layers of BiLSTM and BiGRU to make predictions to obtain GAN-BiRNN.

This article applies these advanced algorithmic changes in the framework of the model proposed in this article, and the structure of the data preprocessing adopted is still the one-hot coding and the physicochemical properties of the features for word2vec segmentation. The results are shown in Table 5:

**Table 5:** Comparison between the model method and advanced algorithms in this article.

Methods	Ts115		CB513		PDB (2018–2020)	
	ACC (Q_3_)	ACC (Q_8_)	ACC (Q_3_)	ACC (Q_8_)	ACC (Q_3_)	ACC (Q_8_)
CNN-LSTM^15^	*90.8%*	**87.9%**	*89.3%*	*84.6%*	96.3%	*95.0%*
Transformer^16^	86.6%	83.6%	83.3%	79.0%	93.9%	92.7%
MLPRNN^37^	87.5%	85.6%	89.2%	84.4%	*96.6%*	**95.3%**
GAN-BiRNN^38^	90.4%	85.5%	88.9%	83.8%	96.1%	94.6%
Distillation-Improved TCN-BiLSTM- MHA	**91.3%**	*87.2%*	**90.3%**	**84.9%**	**96.8%**	**95.3%**

Compared with other models on the same dataset, the model in this article achieves significant performance improvements in terms of the ACC. For the TS115 three-state structure data, the model in this article has a maximum improvement of 4.7% (Transformer), and for its eight-state structure data, it has a maximum improvement of 3.6% (Transformer); for the CB513 three-state structure data, the model in this article has a maximum improvement of 7.0% (Transformer); for its eight-state structure data, it has a maximum improvement of 5.9% (Transformer); for the PDB (2018–2020) three-state structure data, this model has a maximum improvement of 2.9% (Transformer); and for its eight-state structure data, it has up to 2.6% improvement (Transformer).

**Figure 7:**
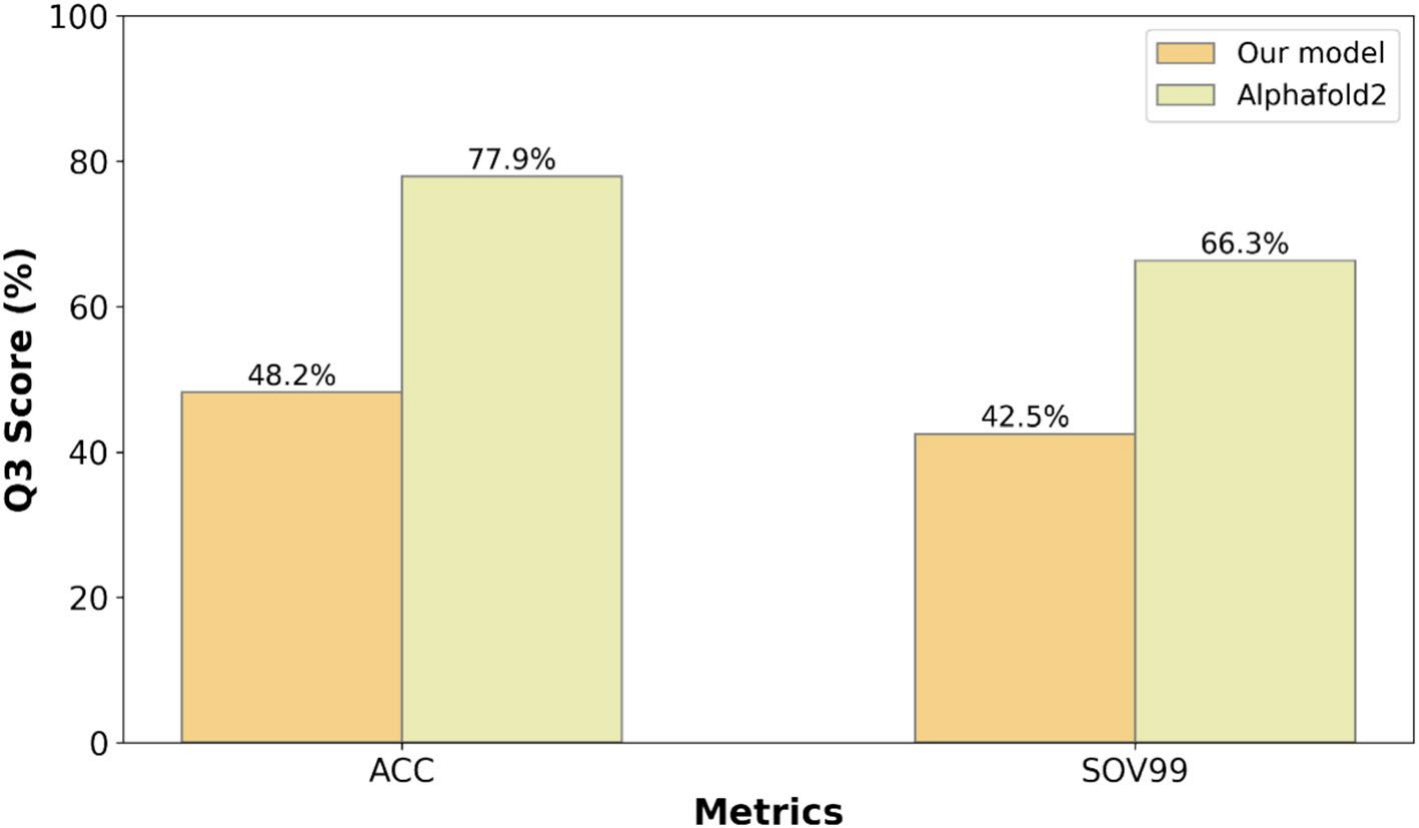
Results of our model and Alphafold2 on the CAMEO-H (2024) test set.

On the other hand, this paper randomly selected 6 protein sequences with low homology sequences in the first half of 2024 from the CAMEO dataset^39^ and used Stride to predict the secondary structure sequence of their pdb files, obtaining the test set CAMEO-H (2024) of this paper. The training set is still PDB (2018–2020). This paper used Alphafold2 and the final model of this paper to predict these parameters, and the results are shown in Fig. 7:

According to Fig. 7, the results of this paper are as follows. The ACC (Q3) and SOV99 (Q3) of our model on the CAMEO-H (2024) test set are 48.2% and 42.5%, respectively. The percentages of Alphafold2 in ACC (Q3) and SOV99 (Q3) were 77.9% and 66.3%, respectively.

(b) Case study

In addition, this paper uses PyMOL to visualize and compare the single-sequence secondary structure data ultimately output by the model and the secondary structure of the original modified sequence to better demonstrate the advantages of the selected comparison model. The visualization data in this paper were selected from protein ID: 1L58 and ID: 1VCA in the RCSB PDB (https://www.rcsb.org/). The prediction models selected are the Transformer, GAN-BiRNN, AlphaFold2, and RGN2 models mentioned in section “Advanced algorithms” and the final model of this paper. In addition, the results of the online protein predictor PSIPRED (http://bioinf.cs.ucl.ac.uk/psipred) were also used. PSIPRED provides a variety of protein structure prediction methods.

**Figure 8:**
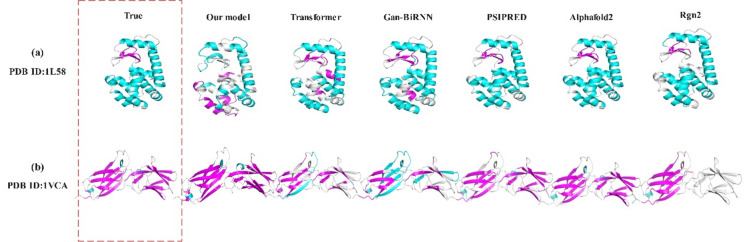
Case study: (**a**) visualization of the amino acid sequence of 1L58 on different models; (**b**) visualization of the amino acid sequence of 1VCA on different models.

The case visualization results are shown in Fig. 8, where cyan blue represents the α-helical structure (H), white represents the irregularly curled structure (C), and fuchsia represents the β-folded structure (E).

As shown in Fig. 8, this article shows that there are better results in the case visualization of the model proposed in this article; on the other hand, Alphafold2 and Rgn2 can also reflect the excellent results of the case visualization and analysis, and this article has the reason that the model proposed in this article has a certain degree of validity; at the same time, the more advanced protein language model can be used well in this problem.

(c) Comparison with other articles

To explore the effectiveness of the model and structure of this article, not only PDB (2018–2020) data but also two classical datasets, TS115 and CB513, are used; therefore, this article reviews the literature on the accuracy and accuracy of Q_8_ and Q_3_ on these two datasets to compare the methods used to optimize the model of this article. Next, this article will briefly introduce these models:

Multiple classifiers^40^: this model processes high-dimensional protein primary sequence data through a two-stage feature selection technique to improve the accuracy of protein secondary structure prediction. The first stage uses unsupervised autoencoders for feature extraction, while the second stage combines three feature selection methods, namely, universal univariate selection, recursive feature elimination, and the Pearson correlation coefficient. By combining these feature selection methods, the model can select the optimal feature subset. Finally, the data based on the selected feature subset are classified using a random forest, decision tree, and multilayer perceptron to achieve protein secondary structure prediction.

MCCM^41^: this model achieves prediction through multilevel feature extraction, a combined classifier module, and a sample difficulty discrimination module. Specifically, the model first introduces a feature extraction module to extract features of different difficulty levels from the data. Then, two classifiers are designed to process simple and difficult samples, where the loss values of difficult samples are weighted to improve the prediction performance of difficult samples. Finally, a sample difficulty discrimination module is designed based on the Dirichlet distribution and information entropy measurement to assign samples to the above classifiers for learning.

AttSec^42^: this model uses the Transformer architecture to capture local features between amino acids through a self-attention mechanism. Specifically, the AttSec model first extracts self-attention maps through a multilayer transformer encoder, which represents pairwise features between amino acid embeddings. These pairwise features are then processed through a two-dimensional convolutional block to detect local patterns. Finally, these local features are converted to one-dimensional features and classified through a fully connected layer to achieve protein secondary structure prediction.

NetSurfP-3.0^43^: this model uses a pretrained protein language model (such as ESM-1b) to generate sequence embeddings and combines a one-dimensional convolutional neural network (1D CNN) and a bidirectional long short-term memory network (BiLSTM) for feature extraction and prediction.

The specific results are shown in Table 6:

**Table 6:** Comparison between the results of our study and those of other studies.

Methods	Ts115		CB513	
	ACC (Q_3_)	ACC (Q_8_)	ACC (Q_3_)	ACC (Q_8_)
Multiple classifiers^40^	–	–	79.0%	**92.0%**
MCCM^41^	–	–	**96.3%**	83.7%
AttSec^42^	*87.5%*	*78.5%*	89.2%	84.4%
NetSurfP-3.0^43^	85.6%	74.9%	84.6%	71.1%
Distillation-Improved TCN-BiLSTM- MHA	**91.3%**	**87.2%**	*90.3%*	*84.9%*

According to the research of existing scholars, the model in this article has better prediction accuracy for Q_8_, but the prediction accuracy for Q_3_ may not be sufficient. For the TS115 three-state structure data, the model in this article has a maximum improvement of 5.7% (NetSurfP-3.0), and for its eight-state structure data, it has a maximum improvement of 12.3% (NetSurfP-3.0). For CB513 three-state structure data, the model in this article has a maximum improvement of 11.3% (multiple classifiers), and for its eight-state structure data, it has a maximum improvement of 1.2% (MCCM).

Additionally, the model in this article uses a less hierarchical structure and simpler construction method. Compared with previous models that use multiple sets and methods, the model in this article is more lightweight and yields relatively excellent results.

(d) Discussion of the results

Compared with all the models mentioned in (a-b) of this section, due to limitations in the size of the data, it is difficult for the model in this article to achieve a significantly better model effect than other models. However, it is worth noting that the experimental results obtained in section “Comparison with other methods”(a) show that if different prediction models are used under the same dataset and data preprocessing method, the model in this article can obtain better results.

On the other hand, this article uses a knowledge distillation algorithm that aims to reduce the computational burden of complex models. Through knowledge distillation, our model learns rich feature representations from large pretrained models while maintaining low computational complexity. This technology not only improves the predictive performance of the model but also significantly reduces the number of parameters in the model, making the model easier to deploy and apply. This article notes that most protein models have problems with large parameters and difficulty in deployment, and our model has made important improvements in this regard.

Compared with other models, the shortcoming of this article is that it does not use more data, and the effect of the model is still limited. However, under the existing datasets and experimental conditions, our model demonstrates excellent performance and high prediction accuracy. Specifically, taking the largest improvement as an example, in the three-state structure prediction of the TS115 dataset, our model has improved by up to 4.7% compared to other models, and in the eight-state structure prediction, it has improved by up to 3.6% compared to other models. In the three-state structure prediction of the CB513 dataset, our model improved by up to 7.0% compared to the other models, and in the eight-state structure prediction, it improved by up to 5.9% compared to the other models. In the three-state structure prediction of the PDB (2018–2020) dataset, in the prediction of the state structure, the model in this article has a maximum improvement of 2.9% compared to the other models, and in the prediction of the eight-state structure, it has a maximum improvement of 2.6% compared to the other models.

Although the limitation of data volume has an impact on the model effect, the innovations in the design and algorithm improvement of the model in this article have made significant progress in terms of prediction performance. Our model has powerful capabilities, especially for processing high-dimensional features and capturing complex dependencies in sequences. In addition, the model's lightweight design and efficient computing architecture give it greater advantages in practical applications.

In short, despite the limitations in data volume, the model in this article performs well in protein secondary structure prediction through clever design and advanced algorithms and has high application value and broad application prospects.

#### Parameter sensitivity analysis

This article proposes Distillation-Improved TCN-BiLSTM-MHA as the final model, which is composed of an improved TCN, BiLSTM-MHA and knowledge distillation, while the improved TCN is divided into three main modules: (1) the TCN model; (2) multimodal fusion; and (3) forward and backward propagation. To show the relationship between performance and the number of scales, this article sets five scales [1], [1,9][1,9,81], [1,9,81,729], and [1,9,81,729,6561] to select the optimal multiscale. At the same time, in the distillation model integrating teacher and student losses for backpropagation and optimization, the alpha coefficients are set, and this article sets the alpha coefficients to 0.1, 0.2, 0.3, 0.4, and 0.5 to determine the optimal alpha coefficients and test the parameter sensitivity. Here, this article chooses the eight-state structure of the Ts115 dataset as the predictor because it has more prediction targets and a smaller dataset, which will be more sensitive to parameter changes.

**Figure 9:**
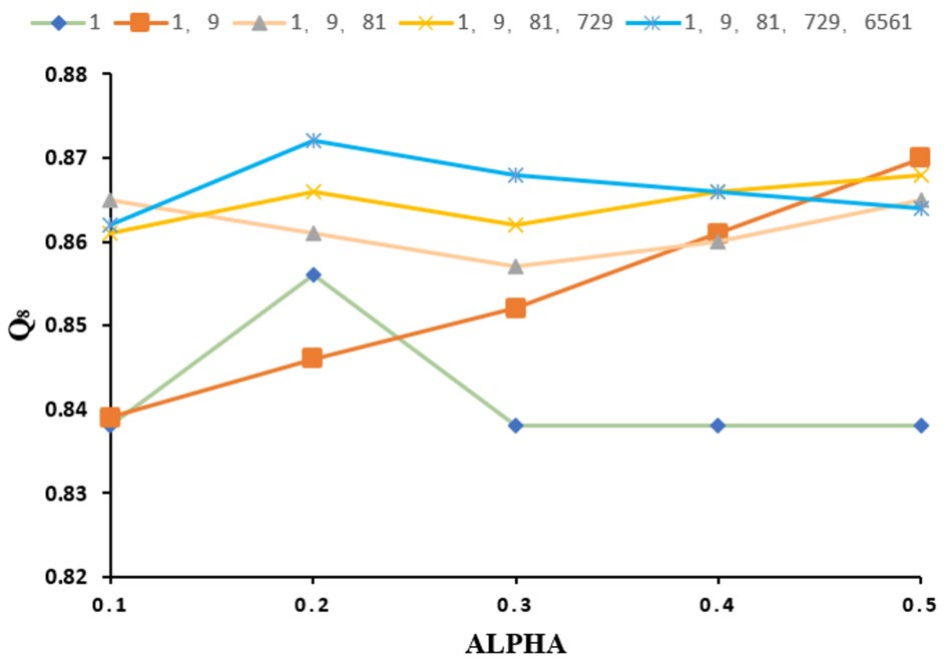
Parameter sensitivity analysis at different scales and alpha coefficients.

As shown in Fig. 9, the model results are obtained in which the scale and alpha coefficients change within the range. This article found that when the scale is [1,9], the change in the alpha coefficient is the most obvious, and the change in Q8 is in the alpha range and reaches a maximum when the coefficient is 0.5; notably, when the scale is 1, the Q8 accuracy is less affected by the alpha coefficient, and its prediction accuracy is the lowest. Therefore, based on sensitivity analysis, this article found that the model fitting effect is best when the scale is [1,9,81,729,6561] and the alpha coefficient is 0.2. Therefore, the scale fixed in this article is [1,9,81,729,6561], and the alpha coefficient is 0.2.

## Conclusions

In this article, an improved TCN-BiLSTM-MHA model combined with knowledge distillation technology is proposed to predict the secondary structure of proteins. The model uses multiscale convolution kernels to capture the local and global dependencies of protein sequences and enhances the understanding of data through bidirectional processing technology. Compared with the traditional unidirectional TCN model, this improved TCN can extract the features of amino acid sequences more comprehensively. The BiLSTM model further captures the global interactions in the sequence through a bidirectional long short-term memory network and combines the multi-head attention mechanism to improve the understanding of complex relationships. On the other hand, this article adopts knowledge distillation technology and uses the ProtT5 pretrained model to guide the student model to learn richer features. The main innovation of this model is that an improved TCN is proposed, and the knowledge distillation model is used to enable the student model to learn the teacher model.

This article uses the TS115, CB513, PDB (2018–2020), CASP13, CASP14, and CASP15 datasets and first uses word2vec technology to extract features and generate word vectors for the primary structural sequences of proteins. For the input features of proteins, one-hot encoding is used to represent the protein sequence, and the physicochemical properties of amino acids are considered to be concatenated as word vectors, which are then input into the improved TCN for feature extraction. Then, the probability is predicted using BiLSTM-MHA, and the final classification result is obtained using the linear model.

The experimental results show that our model has achieved significant improvement in prediction accuracy on the TS115, CB513, and PDB datasets. Compared with the BiLSTM baseline model, the final model in this article has a maximum improvement of 7.9% (CB513) in the eight-state structure data and 8.1% (CB513) in the three-state structure data. Compared with other advanced algorithms, the method in this article has a maximum improvement of 7.9% (CB513 dataset, Transformer model) on three-state structure data and a maximum improvement of 12.3% (CB513 dataset, Transformer model) on eight-state structure data. Specifically, the method in this article not only has advantages in terms of prediction performance but also contributes to the structural optimization and feature extraction of the model, thereby achieving more efficient protein secondary structure prediction.

Word2vec is a natural language processing method that is not widely used in protein secondary structure prediction at present. However, in this article, the results are somewhat improved compared to those of other scholars' language processing work; much of the current scholars' work, i.e., directly inputting existing fusion features rather than obtaining embedded word vectors^42^; and at the same time, the use of knowledge distillation to learn increasingly effective features from an existing, complex and feature-rich protein language model that has already learned features has been proven to be effective in this article. to be effective. The model structure proposed in this article has obtained better results on classical datasets, which indicates that its structure is somewhat reasonable and suitable for this topic. To this end, the following conclusions are drawn: (1) the natural language processing of word vector feature embedding as a protein input sequence using word2vec has a certain degree of effectiveness; (2) the new model structure of knowledge distillation learning proposed in this article, improved TCN-BiLSTM-MHA, is effective, and the prediction of the protein secondary structures of eight-state and three-state can obtain good results; and (3) the knowledge distillation learning proposed in this article is effective. (4) Knowledge distillation learning for the model structure in this article effectively enhances the results for small datasets.

However, for the structure and model proposed in this article, there are still problems and improvement directions to be explored: (1) A better protein language model can be selected for knowledge distillation. Currently, although the protein language learns rich features, the accuracy of its model is not high enough, and a protein language model with higher accuracy can be selected under better configuration and arithmetic. (2) Features can be extracted in a rich manner according to different protein learning tasks. In this article, the one-hot coding and physicochemical properties of proteins are selected as feature inputs. However, regarding the features of protein sequences, many scholars have studied PSSMs, and all kinds of PSSMs encoding PSSMs have produced different results for different protein sequence features^32^. (3) The models can be improved. For protein secondary structure prediction, there are also methods such as the use of transformers that can better help the prediction in this article, and additional novel structural and modeling methods can be further explored.

## Data Availability

The data and code associated with this study have been deposited at https://github.com/riguangliunian/Protein-structure-prediction.
